# Correction: Validation of Serological Tests for the Detection of Antibodies Against *Treponema pallidum* in Nonhuman Primates

**DOI:** 10.1371/journal.pntd.0003757

**Published:** 2015-04-27

**Authors:** The PLOS Neglected Tropical Diseases Staff

## Notice of Republication

This article was republished on April 10, 2015 to correct an error in the sizing of Figure 1 that was introduced during the production process. The publisher apologizes for the error. Please download this article again to view the correct version. The originally published, uncorrected article and the republished, corrected article are provided here for reference.

## Supporting Information

S1 File Originally published, uncorrected article. (PDF)

S2 File Republished, corrected article. (PDF)
