# Supplementary material for: Correction: Validation of Serological Tests for the Detection of Antibodies Against Treponema pallidum in Nonhuman Primates
Source: PLoS Negl Trop Dis. 2015 Apr 27;9(4):e0003757. doi: 10.1371/journal.pntd.0003757 (PMC4411047; doi:10.1371/journal.pntd.0003757)
Supplement: S1 File — (PDF) [file pntd.0003757.s001.pdf]

PLOS | NEGLECTED TROPICAL DISEASES

RESEARCH ARTICLE

Validation of Serological Tests for the Detection of Antibodies Against *Treponema pallidum* in Nonhuman Primates

Sascha Knauf[1]*, Franziska Dahlmann[1], Emmanuel K. Batamuzi[2], Sieghard Frischmann[3], Hsi Liu[4]

1 German Primate Center, Pathology Unit, Work Group Neglected Tropical Diseases, Göttingen, Germany, **2** Sokoine University of Agriculture, Faculty of Veterinary Medicine, Department of Surgery and Theriogenology, Morogoro, Tanzania, **3** Mast Diagnostica GmbH, Reinfeld, Germany, **4** National Center for HIV/AIDS, Viral Hepatitis, STD, and TB Prevention, Centers for Diseases Control and Prevention, Atlanta, Georgia, United States of America

* sknauf@dpz.eu

CrossMark
click for updates

OPEN ACCESS

Citation: Knauf S, Dahlmann F, Batamuzi EK, Frischmann S, Liu H (2015) Validation of Serological Tests for the Detection of Antibodies Against *Treponema pallidum* in Nonhuman Primates. PLoS Negl Trop Dis 9(3): e0003637. doi:10.1371/journal.pntd.0003637

Editor: Mathieu Picardeau, Institut Pasteur, FRANCE

Received: November 21, 2014

Accepted: February 22, 2015

Published: March 24, 2015

Copyright: This is an open access article, free of all copyright, and may be freely reproduced, distributed, transmitted, modified, built upon, or otherwise used by anyone for any lawful purpose. The work is made available under the Creative Commons CC0 public domain dedication.

Data Availability Statement: All relevant data are within the paper and its Supporting Information files.

Funding: SK received funding and support for the field work at Lake Manyara National Park from German Academic Exchange Program (D/06/43974, https://www.daad.de/de/), Christian Vogel Fond (2006, http://www.gf-primatologie.de/), World Association Zoos and Aquariums (07002, http://www.waza.org/en/site/home), University of Leipzig, Justus Liebig University of Giessen, Scil animal care company, Telinject, Translogistic, and Wuppertal Zoo. The funders had no role in study design, data

Abstract

There is evidence to suggest that the yaws bacterium (*Treponema pallidum* ssp. *pertenue*) may exist in non-human primate populations residing in regions where yaws is endemic in humans. Especially in light of the fact that the World Health Organizaiton (WHO) recently launched its second yaws eradication campaign, there is a considerable need for reliable tools to identify treponemal infection in our closest relatives, African monkeys and great apes. It was hypothesized that commercially available serological tests detect simian anti-*T. pallidum* antibody in serum samples of baboons, with comparable sensitivity and specificity to their results on human sera. Test performances of five different treponemal tests (TTs) and two non-treponemal tests (NTTs) were evaluated using serum samples of 57 naturally *T. pallidum*-infected olive baboons (*Papio anubis*) from Lake Manyara National Park in Tanzania. The *T. pallidum* particle agglutination assay (TP-PA) was used as a gold standard for comparison. In addition, the overall infection status of the animals was used to further validate test performances. For most accurate results, only samples that originated from baboons of known infection status, as verified in a previous study by clinical inspection, PCR and immunohistochemistry, were included. All tests, TTs and NTTs, used in this study were able to reliably detect antibodies against *T. pallidum* in serum samples of infected baboons. The sensitivity of TTs ranged from 97.7-100%, while specificity was between 88.0-100.0%. The two NTTs detected anti-lipoidal antibodies in serum samples of infected baboons with a sensitivity of 83.3% whereas specificity was 100%. For screening purposes, the TT Espline TP provided the highest sensitivity and specificity and at the same time provided the most suitable format for use in the field. The enzyme immune assay Mastblot TP (IgG), however, could be considered as a confirmatory test.

collection and analysis, decision to publish, or preparation of the manuscript.

Competing Interests: I have read the journal's policy and the authors of this manuscript have the following competing interests: SF is employed by Mast Diagnostica GmbH, a company that sells treponemal tests (Serodia TP-PA, Espline TP, Mastafluor FTA-ABS IgG, and Mastablot TP IgG) that were used in this study. ALL.DIAG S.A.S (http://www.alldiag.com/) provided Syphilitop Optima tests. This does not alter our adherence to all PLOS policies on sharing data and materials.

Author Summary

The success of any disease eradication campaign depends on considering possible non-human reservoirs of the disease. Although the first report of *T. pallidum* infection in baboons was published in the 1970's and the zoonotic potential was demonstrated by inoculation of a West African simian strain into humans, nonhuman primates have not yet been considered as a possible reservoir for re-emerging yaws in Africa. Simian strains are genetically most closely related to the strains that cause yaws in humans. The identification of baboons as a reservoir for human infection in Africa would be revolutionary and aid important aspects to yaws eradication programs. Reliable serological tests and a useful standardized test algorithm for the screening of wild baboon populations are essential for studying potential transmission events between monkeys and humans.

Introduction

Treponema pallidum is the bacterium that causes venereal syphilis (ssp. *pallidum*) and the non-venereal diseases yaws (ssp. *pertenue*) and endemic syphilis (ssp. *endemicum*) in humans [1]. The spirochete is able to cause a life-long chronic infection in untreated individuals [2] and elicits a strong adaptive immune response against a wide array of antigens [3–4] with strong serum IgM and IgG response [5–7] towards a number of lipoproteins (e.g., Tp15, 17, and 47), endoflagellar proteins (e.g., FlaA, FlaB1, 2, and 3), and the Tpr family proteins (e.g., TprA-TprL) [6]. Furthermore, infection-related cellular damage is known to induce the production of non-treponemal antibodies mainly directed against cardiolipids [8,9].

Recently, we have reported that *T. pallidum* can infect large numbers of African monkeys and great apes [10]. To date, all simian isolates seem to be closely related to *T. pallidum* ssp. *pertenue*, the pathogen causing human yaws [11,12] and at least the Fribourg-Blanc simian strain, which was isolated from a baboon in Guinea [13], has the potential to cause sustainable infection in humans [14]. Thus, there is evidence to suggest that yaws exists in non-human primate populations residing in regions where humans are also infected [15]. The clinical manifestations in non-human primates (NHPs) however, show regional differences. While West African simian strains of *T. pallidum* mostly cause no clinical signs [16], gorillas in the Republic of the Congo show yaws-like lesions [17] and baboons in East Africa are known to develop severe genital ulceration [11,18]. However, independent of the clinical manifestations simian strains induce a pronounced serological response in the respective host [10], which may be used to screen and identify host populations for their potential as a natural reservoir.

In the context of the possible zoonotic potential of simian strains [14], the identification and knowledge of a nonhuman reservoir for *T. pallidum* is crucial to disease elimination or eradication efforts and could help to identify hot spots for potential simian-to-human disease transmission. There is therefore considerable need to validate treponemal tests (TTs) and non-treponemal (NTTs) for their use in NHPs. Due to the close relationship of simian and human treponemes [12], we hypothesized that A) commercially available serological tests are able to detect simian anti-*T. pallidum* IgM and IgG in serum samples of baboons, a NHP species with high infection rates and B) that the serological tests will be equally reliable in terms of sensitivity and specificity in baboon sera compared to the human sera.

Materials and Methods

Ethics statement

Baboon serum samples were taken in accordance with the Tanzania Wildlife Research Institute's Guidelines for Conducting Wildlife Research (2001) and with permission of Tanzania National Parks (TNP/HQ/E.20/08B) as well as Commission for Science and Technology in Tanzania (2007-56-NA-2006-176). The committee of Tanzania National Parks and Tanzania Wildlife Research Institute approved sample collection. Baboon serum samples from the German Primate Center were granted from the institute's bio bank and originated from healthy animals that were sampled during post-mortem examination. The Animal Welfare and Ethics Committee of the German Primate Center approved the use of samples for this study.

Study site and animals

In a previous study, we were able to detect *T. pallidum* infection in wild olive baboons (*Papio anubis*) at Lake Manyara National Park in Tanzania [18]. Although the isolated strain is most closely related to *T. pallidum* ssp. *pertenue* [11], the pathogen causes severe genital ulceration. Diagnosis was based on gross pathology, histology, and molecular biological tests. The latter included quantitative [19] and qualitative PCR [20], targeting the *polA* gene of *T. pallidum*. DNA was extracted from skin tissue samples [18]. Data and corresponding serum samples that were constantly stored at -80°C of 57 untreated baboons from this study were available for analysis in 2013. An additional set of 11 serum samples of healthy captive olive baboons from the German Primate Center were included as negative control. The extent of genital ulceration was used to classify and group animals as clinically healthy, initially-, moderately-, or severely-infected (Fig. 1). It is not known whether simian infection develops in stages similar to human infection.

Treponemal tests (TTs)

***Serodia TP-PA* (Fujirebio Diagnostics Inc., Malvern, PA, USA; Cat. No. 201326).** The test uses sensitized colored gelatin particles as carriers of the *T. pallidum* (Nichols Strain) antigen and is run in microtiter plate reaction wells (high-binding and U-shaped, Cat. No. 650061, Greiner bio-one, Frickenhausen, Germany). All steps and control standards followed the manufacturer's protocol. Serum was not heat pre-treated, but sera that showed agglutination with unsensitized and sensitized gelatin particles were re-tested with a pre-absorption step as recommended by the manufacturer. A quantitative analysis was carried out using an initial titration scheme (1:5 to 1:20,480). Serum samples showing high reaction titers of $\geq$ 1:20,480 were re-tested until an end-point with negative reaction was reached. Results were interpreted in accordance with the manufacturer's protocol. Two independent investigators read each test result on the day of testing and in addition 24 hours later. Performance characteristics as communicated by the manufacturer are summarized in Table 1. All baboon serum samples were tested at least twice. In this study and in accordance with the European Guidelines on the Management of Syphilis [21–23], *T. pallidum* particle agglutination assay (TP-PA) was used as the initial screening test and gold standard to be compared to all other test performances.

***Espline TP* (Fujirebio Diagnostics Inc., Malvern, PA, USA; Cat. No. 219126).** This screening test comes in a ready-to-use cassette format and is licensed for use in serum or plasma samples. It is based on immunochromatography for the detection of IgM and IgG class antibodies to *T. pallidum*. The recombinant antigens Tp47 (0.7 μg/cassette), Tp15-17 fusion protein (0.4 μg/cassette), alkaline phosphatase labeled TP recombinant antigen (Tp 47, 17.0 ng/cassette), and alkaline phosphatase labeled TP recombinant antigen (Tp15-17, 14.8 ng/

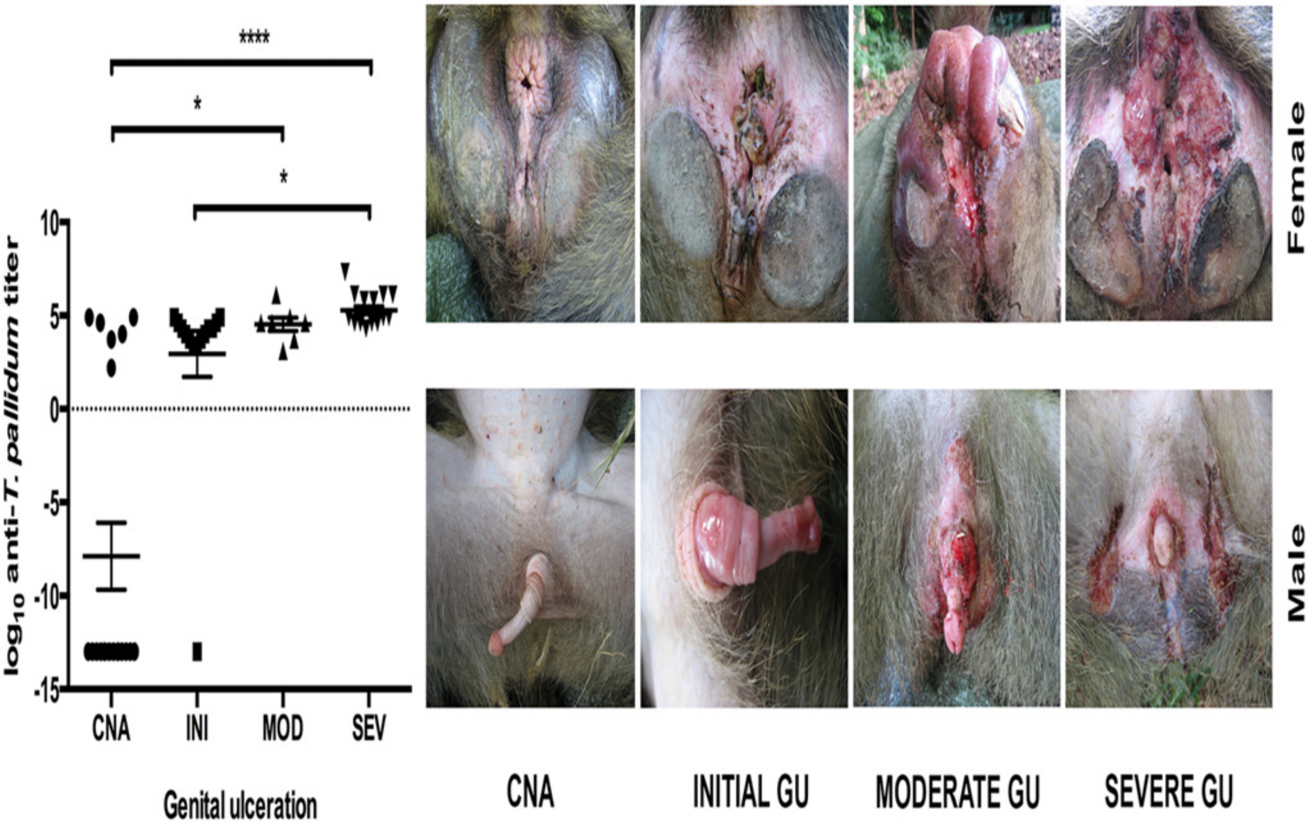

Fig 1. Multiple comparisons of log anti-*T. pallidum* titers in 4 groups with a different stage of genital ulceration in baboons ([CNA] = clinically non-affected (n = 20), [INI] = initial (n = 14), [MOD] = moderate (n = 7), and [SEV] = severe genital ulceration (n = 16); for stage definition see [18]), GU = genital ulceration. Anti-*T. pallidum* antibody quantification was investigated using the Serodia TP-PA. Kruskal-Wallis test using Dunn's correction for multiple comparison: CNA vs. SEV mean rank diff. = -30.04, $p \leq 0.0001$; CNA vs. MOD mean rank diff. = -19.95, $p \leq 0.05$; INI vs. SEV mean rank diff. = -17.56, $p \leq 0.05$. (mean ± SEM).

doi:10.1371/journal.pntd.0003637.g001

cassette) were coated on the reaction membrane. The assay was operated and interpreted according to the manufacturer's instructions. Test results were considered valid when the internal reference line was present. All baboon serum samples were tested at least twice and were not heat pre-treated.

***Syphilitop Optima* (ALL. DIAG S.A.S., Strasbourg, France, Cat. No. 5480).** According to the manufacturer's protocol, this immunochromatographic test must only be used on serum. Its membrane is partially coated with a mixture of different *T. pallidum*-specific antigens (17 kDa and 47kDa). Serum migrates along the test strip through chromatography. IgM and IgG class anti-*T. pallidum* antibodies bind to the antigen(s), indicated by the appearance of purple-colored indicator bands. Only tests that showed an internal reference band were

Table 1. Performance characteristics of the serological tests used in this study, as reported by the manufacturer. Sen = Sensitivity, Spec = Specificity, n.p. = not provided.

	Serodia TP-PA (n)	Espline TP (n)	Syphilitop Optima (n)	Mastafluor FTA-ABS IgG (n)	Mastablot TP IgG (n)	VDRLCHECK CHARBON/ RPR (n)	RPR-100 (n)
**Sen**	100.0% (145)	100.0% (145)	100.0% (103)	n.p.	n.p.	100.0% (50)	n.p.
**Spec**	100.0% (935)	99.7% (932)	95.0% (100)	n.p.	n.p.	100.0% (100)	n.p.

doi:10.1371/journal.pntd.0003637.t001

considered for valid test interpretation. All baboon serum samples were tested at least twice and were not heat pre-treated.

***Mastafluor FTA-ABS* IgM and IgG (Mast Diagnostica, Reinfeld, Germany; Cat. No. 6305222).** The Fluorescence-*T. pallidum*-Antibody-Absorption-Test (FTA-ABS) kit for IgM and IgG was used to test serum samples of baboons. Samples were not heat pre-treated. Depending on the conjugate antibody, it was possible to distinguish between IgM or IgG class antibodies. The test procedure followed the manufacturer's protocol.

***Mastablot TP* (Mast Diagnostica, Reinfeld, Germany).** The immunoblots Mastablot TP IgM (Cat. No. 6653M24) and IgG (Cat. No. 6653G08) were used to detect anti-*T. pallidum* antibodies in all serum samples of baboons. Both blots use nitrocellulose strips coated with p15, p17, TmpA and p47. Each immunoblot was used according to the manufacturer's guidance. As in all tests, serum was used without heat pre-treatment.

Non-treponemal tests (NTTs)

***VDRLCHECK CHARBON/RPR* (ALL. DIAG S.A.S., Strasbourg, France; Cat. No. 5474).** This macroscopic non-treponemal flocculation test for the detection, and to a certain extent quantification of anti-lipoidal antibodies was used in all serum samples of baboons. Tests were repeated twice and serum samples were not heat pre-treated. Serum was mixed with the VDRLCHECK CHARBON/RPR reagent and allowed to react for eight minutes. In case anti-lipoidal antibodies were present, black macroscopic visible floccules were visible. The test procedure and test interpretation was operated according to the manufacturer's protocol. To avoid false-negative test results due to the prozone phenomenon [24], critical test results were retested with 1:8 dilution.

***RPR-100* (Biorad, Marnes, France; Cat. No. 72505).** Similar to the VDRLCHECK CHARBON/RPR Test, the Rapid Plasma Reagin (RPR) test kit was used in serum samples of baboons to detect qualitative and semi-quantitative IgM and IgG antibodies against lipoidal material that originates from host cell damage or lipoprotein-like particles of the spirochete. Available samples were tested twice. The test procedure and interpretation of results were based on the manufacturer's protocol. The presence of antibodies resulted in macroscopic visible agglutination of carbon-particles coated with a mix of liquid antigens. Carbon particles are dispersed in a medium that contains not otherwise specified substances to eliminate unspecific reaction. To avoid false-negative test results due to the prozone phenomenon [24], critical test results were retested with 1:8 dilution.

At least one researcher and an experienced technician read the test results of each test. Results of each test were blinded and were communicated independently. If not otherwise specified, each test was performed on a single day. Performance characteristics of all tests, as reported by the manufacturer, are summarized in Table 1.

Definition of test results

Generally, the definition of a test result was based on the individual's overall infection status (Table 2). Details of infection status including genital ulceration status and each test's interpretation can be found in the Supporting Information (S1 Table).

True positive (Tpos). For TTs, a true positive test result was assumed, when the result coincided with the majority of all other serological test results of TTs, which were positive (3 out of 5, excluding tests to detect anti-*T. pallidum* IgM), a positive outcome of the skin tissue PCR, and/or immunohistochemistry (IHC). NTTs were considered Tpos when molecular biological tests indicated the presence of *T. pallidum* and/or when the test result correlated to the overall outcome of the TT results.

Table 2. Definitions used to determine the infectious stage of baboons.

Test		Results				
Genital Ulceration	/	+/-	+/-	-	+/-	-
IHC	/	+ (1 of 2)	-	-	-	-
PCR						
**Treponemal Tests**	/	+ (1 of 2)	-	-	-	-
Serodia TP-PA	3 of 5 tests	+/-	+	-	+	-
Espline TP						
Syphilitop Optima	3 of 5 tests	+/-	+	-	+	-
Mastafluor FTA-ABS IgG	3 of 5 tests	+/-	+	-	+	-
Mastablot TP IgG	3 of 5 tests	+/-	+	-	+	-
**Non-Treponemal Tests**	3 of 5 tests	+/-	+	-	+	-
VDRLCHECK CHARBON/RPR	/	+/-	+	+	-	-
RPR-100		+/-	+	+	-	-
**Infection Status**	/	**+**	**+**	**-**	**+**	**-**

The definition of infection status was based on the outcome of serological (this study) and molecular biological tests [18, S1 Table].

doi:10.1371/journal.pntd.0003637.t002

False positive (Fpos). A false positive test result of TTs was presumed when the outcome of a test was contrary to all other test results of the TTs, which were negative (3 out of 5, excluding tests to detect anti-*T. pallidum* IgM), as well as a negative skin tissue PCR and IHC result. NTTs were considered Fpos when molecular identification and the second NTT were negative.

True negative (Tneg). A TT result was defined as true negative when it conformed to the majority of all other TT results (3 out of 5, excluding tests to detect anti-*T. pallidum* IgM) along with a negative outcome of the skin tissue PCR and IHC. NTTs were defined Tneg, when both NTTs were non-reactive, while the overall TT result could be positive or negative.

False negative (Fneg). False negative TT results were documented when a result was contrary to all other test results of the serological tests, which were positive (3 out of 5, excluding tests to detect anti-*T. pallidum* IgM), along with a positive skin tissue PCR and/or IHC result. NTTs were considered Fneg, when molecular biological tests indicated the presence of the spirochete and one of the second NTTs, VDRL or RPR, became reactive.

Statistics

Statistical analyses were performed using Prism 6.0 (GraphPad Software). Results of the TTs were first compared to the result of the Serodia TP-PA and second to the consensus of infection status, as it is defined in Table 2, and which takes into account the appearance of clinical symptoms (genital health status), IHC and skin tissue PCR results of the same animals as published elsewhere [18]. While the TP-PA was used as the gold standard for TTs, we compared results to the baboon's infection status for further verification of test results and accuracy. With regard to NTTs it was assumed that a significant proportion of tests might become nonreactive in chronically infected baboons, as it is described for untreated human syphilis infection [25–27]. Test performances of the NTTs were therefore evaluated exclusively on the basis of the consensus of infection.

A non-parametric test for nominal scale data, the two-tailed Fisher's Exact Test, was used to compare the proportions among the serological tests, skin tissue PCR results and clinical signs of infection as well as for the analysis of sensitivity and specificity of the serological tests.

Sensitivity. Proportion of actual positives that are correctly identified as true positives: Tpos/(Tpos + Fneg).

Specificity. Proportion of negative test results that are correctly identified as being negative: Tneg/(Tneg + Fpos).

Efficiency. Proportion of true test results within the overall sample size for the test: (Tpos + Tneg)/n total.

Positive and negative predictive value. Probability of a positive/negative test result to be true positive/negative: Tpos/(Tpos + Fpos) and Tneg/(Tneg + Fpos).

Endpoint titers of exponential scale were log_{10}-transformed to reduce variance. In case of non-Gaussian distribution and log_{10}-transformation, zero-titers were converted into 10e-14. Normal distribution was tested using the D'Agostino & Pearson omnibus normality test and the Shapiro-Wilk normality test. Antibody titers were analyzed using four different baboon categories: clinically non-affected, initial, moderate, and severe stage of the genital ulceration as it was documented in the field [18]. The non-parametric ANOVA using Krustal-Wallis test was applied to the log_{10}-transformed data sets of the antibody titers. Each mean rank of a genital ulceration stage (clinically non-affected [CNA], initial [INI], moderate [MOD], and severe genital-ulcerated [SEV]) was compared to mean rank of every other genital ulceration stage. Dunn's correction for multiple comparisons (significance without confidence intervals) was applied to the test. In all tests, $p \leq 0.05$ was considered statistically significant.

Results

Five treponemal and two non-treponemal tests were evaluated

Tables 3 and 4 summarize sample size, proportions, and performance characteristics of the serological tests that were used in the 57 baboon serum samples from Lake Manyara National Park and an additional set of 11 serum samples from olive baboons of the German Primate Center in Germany. The latter were included for the purpose of additional negative control.

Table 3. Comparison of treponemal serological tests with the results of the Serodia TP-PA.

Assay and Result	Serodia TP-PA		p-Value	% Sensitivity (95% CI)	% Specificity (95% CI)	% Pos. pred. value (95% CI)	% Neg. pred. value (95% CI)
	Pos.	Neg.					
**Treponemal Tests**							
**Espline TP**							
Positive	42	1	< 0.0001	97.7 (0.877–0.999)	96.0 (0.797–0.999)	97.7 (0.877–0.999)	96.0 (0.797–0.999)
Negative	1	24					
**Syphilotop Optima**							
Positive	42	4	< 0.0001	91.3 (0.792–0.976)	95.5 (0.772–0.999)	97.7 (0.877–0.999)	84.0 (0.639–0.955)
Negative	1	21					
**Mastafluor FTA-ABS IgG**							
Positive	42	1	< 0.0001	97.7 (0.877–0.999)	96.0 (0.796–0.999)	97.7 (0.877–0.999)	96.0 (0.796–0.999)
Negative	1	24					
**Mastablot TP IgG**							
Positive	41	0	< 0.0001	100.0 (0.914–1.000)	94.7 (0.740–0.999)	97.6 (0.874–0.999)	100.0 (0.815–1.000)
Negative	1	18					

Two-tailed Fisher's exact test. Pos. pred. value = positive predictive value, Neg. pred. value = negative predictive value.

doi:10.1371/journal.pntd.0003637.t003

Table 4. Comparison of the serological tests with the consensus of infection status (Table 2).

Assay and Result	Serodia TP-PA	Espline TP	Syphilitop Optima	Mastafluor FTA-ABS IgG	Mastablot TP IgG	VDRL	RPR-100
n Tpos	42	43	43	43	41	10	10
n Fpos	2	0	3	0	0	0	0
n Tneg	23	25	22	25	19	27	27
n Fneg	1	0	0	0	0	2	2
n Total	68	68	68	68	60	39	39
% Sensitivity (95% CI)	97.7 (0.877–0.999)	100.0 (0.918–1.000)	100.0 (0.918–1.000)	100.0 (0.918–1.000)	100.0 (0.914–1.000)	83.3 (0.516–0.979)	83.3 (0.516–0.979)
% Specificity (95% CI)	92.0 (0.740–0.990)	100.0 (0.863–1.000)	88.0 (0.688–0.975)	100.0 (0.863–1.000)	100.0 (0.824–1.000)	100.0 (0.872–1.000)	100.0 (0.872–1.000)
% Pos. pred. value (95% CI)	95.5 (0.845–0.994)	100.0 (0.918–1.000)	93.5 (0.821–0.986)	100.0 (0.918–1.000)	100.0 (0.914–1.000)	100.0 (0.692–1.000)	100.0 (0.692–1.000)
% Neg. pred. value (95% CI)	95.8 (0.789–0.999)	100.0 (0.863–1.000)	100.0 (0.846–1.000)	100.0 (0.863–1.000)	100.0 (0.824–1.000)	93.1 (0.772–0.992)	93.1 (0.772–0.992)
% Efficiency	95.6	100.0	95.6	100.0	100.0	94.9	94.9

Two-tailed Fisher's exact test in all serology tests, $p < 0.0001$. Pos. pred. value = positive predictive value, Neg. pred. value = negative predictive value. Sample size differs because in some cases sample material was on short supply for further tests. For the NTTs only serum was tested and included here. Tests in all other animals were preformed with plasma and can be found in S2 Table.

doi:10.1371/journal.pntd.0003637.t004

Treponemal and non-treponemal tests detect antibodies against *T. pallidum* in serum samples of infected baboons

When comparing TT performances with the TP-PA, we observed test sensitivity in the range of 91.3–100%, and specificity ranging from 94.7–96.0% (Table 3). When test results were compared to the consensus of all test results including PCR, however, the observed sensitivity of the TTs was in the range of 97.7–100%; whereas the specificity reduced slightly to the range of 88.0–100% (Table 4). This reduction was almost exclusively caused by the test performance of Syphilitop Optima (Table 4). NTT performances were not compared to TP-PA results since positive TT results in untreated, chronically infected patients do not necessarily predict reactivity of the corresponding NTT [25–27]. However, both NTTs used in this study reliably detected anti-lipoidal antibodies in serum (Table 4) or plasma samples (S2 Table) of baboons. When infection status was considered in the context of all test results including PCR analysis, NTT sensitivity in serum samples was lower (83.3%) than the average of the TTs (99.54%). The specificity of VDRL and RPR in serum samples was 100% and therefore higher than the performance of the standard TP-PA (92.0%) and Syphilitop-Optima rapid test (88.0%). The performance data of NTTs for plasma samples are listed in S2 Table.

Anti-*T. pallidum* antibodies were found in 97.3% of baboons with genital ulceration and in 6 of 20 animals that were clinically healthy (30.0%, Table 5, Fig. 1). For comparison, a remarkable proportion of genital-ulcerated baboons (13.5%; n = 5/37, Table 5) had a negative PCR outcome of their respective skin tissue biopsy. Vice versa, we found 30% (n = 6/20) genital non-genital-ulcerated baboons with positive *T. pallidum* PCR of their corresponding skin biopsy. Yet, genital-ulcerated baboons were significantly more often reactive for *T. pallidum* in serology (TTs, $p < 0.0001$) and skin tissue PCR ($p < 0.0001$) compared to clinically healthy and thus non-ulcerated baboons. Both treponemal rapid tests, Espline TP and Syphilitop Optima, were more sensitive than the Serodia TP-PA (Two-tailed Fisher's exact test, $p < 0.0001$, Table 4), although specificity in the Syphilitop Optima was much lower than Serodia TP-PA. The same applied, when Mastafluor FTA-ABS IgG and Mastablot TP IgG were compared to the Serodia TP-PA particle agglutination assay. In both tests the proportion of positive results matched the results of the Serodia TP-PA (Two-tailed Fisher's exact test, $p < 0.0001$, Table 4).

Table 5. Crosstab of the results obtained from 57 baboon and 11 control samples.

Genital Ulceration	CNA		Initial		Moderate		Severe		Negative Control	
n	20		14		7		16		11	
	+	-	+	-	+	-	+	-	+	-
PCR	6	14	10	4	7	0	15	1	n/t	n/t
IHC	2	18	3	11	4	3	7	9	n/t	n/t
Serodia TP-PA	8	12	13	1	7	0	16	0	0	11
Espline TP	6	14	14	0	7	0	16	0	0	11
Syphilitop Optima	9	11	14	0	7	0	16	0	0	11
Mastafluor FTA-ABS IgG	6	14	14	0	7	0	16	0	0	11
Mastablot TP IgG	6	8	12	0	7	0	16	0	0	11
VDRL (Serum)	2	7	1	7	2	3	5	1	0	11
RPR (Serum)	2	7	2	6	2	3	4	2	0	11

CNA = clinically non-affected.

doi:10.1371/journal.pntd.0003637.t005

Anti-*T. pallidum* IgM antibodies were not detected

No correlation was found when anti-*T. pallidum* IgG positive serum samples were tested in the immunoblot assay Mastablot TP for the presence of IgM antibodies against *T. pallidum*. Only a limited number of animals, 6 out of 59 (10.2%), tested positive for both anti-*T. pallidum* IgG and IgM antibodies. We did not find any samples that were positive for anti-*T. pallidum* IgM only.

Severe genital ulceration is associated with high antibody titers

Even after $\log_{10}$-transformation of antibody titers, the clinically healthy and the initial stage genital-ulcerated group (Fig. 1; it is not known whether NHPs develop three stages similar to humans; initial stage refers to the severity of genital ulceration as describe elsewhere [18]) were not normally distributed. The Kruskal-Wallis test using Dunn's correction for multiple comparisons showed that antibody titers in the severe genital-ulcerated group of baboons were significantly higher when compared to clinically healthy animals (mean rank diff. = -30.04, $p \leq 0.0001$) and baboons with an initial stage of genital ulceration (mean rank diff. = -17.56, $p \leq 0.05$). The same was found for moderate genital-ulcerated baboons, which had significantly higher antibody titers against *T. pallidum* than animals without genital ulceration (mean rank diff. = -19.95, $p \leq 0.05$). Fig. 1 provides an overview.

Discussion

All serological tests used in this study, TTs as well as NTTs, were able to detect anti-*T. pallidum* antibodies in serum of infected baboons. While the presence of anti-*T. pallidum* IgG antibodies in all infected animals correlates to lifelong antibody titers in human infection [28], the absence of IgM type antibodies against the spirochete in infected baboons (only 6 animals were positive for anti-*T. pallidum* IgM antibodies, Mastablot IgM, S1 Table) could most likely be linked to the timing of sampling from two weeks to months post infection. This may not be fully consistent with human cases, in which IgM antibody titers are low to moderately high in primary syphilis, peak in secondary and sometimes tertiary syphilis, and are low in latent syphilis [29]; the majority of baboons, which were tested in this study were unlikely in a latent stage of infection. Although animals are known to be chronically infected and untreated over many months

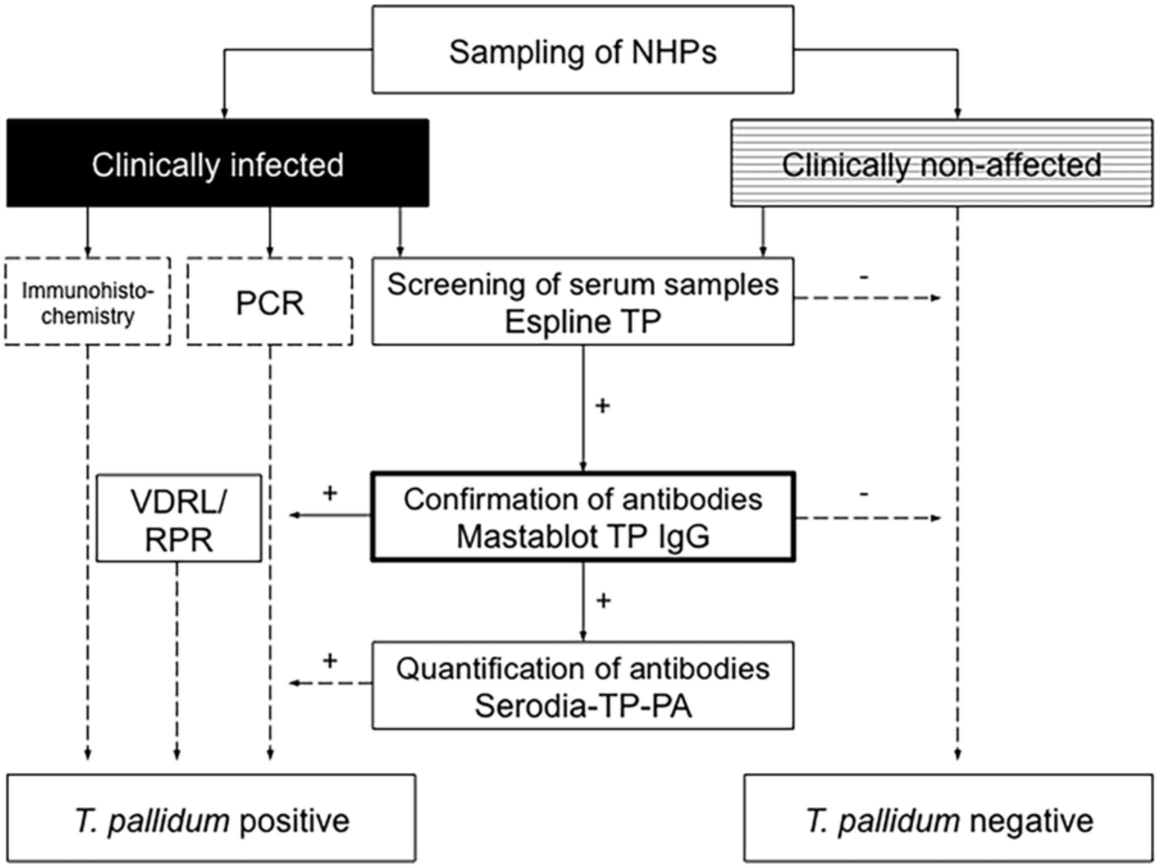

Fig 2. *T. pallidum* test algorithm for the screening of wild non-treated baboons. Based on the test performances Espline TP is recommended as the initial screening test followed by a confirmatory test e.g. Mastablot TP IgG that has been identified as most reliable standard. Dashed lines indicate reported results, while continuous lines represent the workflow.

doi:10.1371/journal.pntd.0003637.g002

to years, genital ulceration in infected baboons rarely heals up. However, the expected lower amplitude of IgM titers [30] and the expected reduced half-life of IgM [31] may have contributed to the result. Results were excluded from multiple comparisons analysis and evaluation of performance characteristics, because of the uncertainty and low number of IgM positive samples. The finding of few chronically infected individuals positive for IgM antibodies against *T. pallidum* could be explained by persisting IgM titers, as described for other human spirochete infections (e.g., borreliosis) [32,33].

All TTs and NTTs used in this study are commercially available and licensed for use with human serum or plasma samples. Using TT as a screening test in NHPs (Fig. 2) is in accordance with international standards and EU Guidelines for the Management of Syphilis in humans [22,34,35] and is often referred to as "reverse testing algorithm" [36]. Although there were no data about efficiency, sensitivity, and specificity available from testing samples in baboons or other NHPs, it was reasonable to assume that a TP-PA would be a reliable standard in testing baboon serum samples both qualitatively and quantitatively (Table 3). A number of alternative TTs (Espline TP, Syphilitop Optima, Mastafluor FTA-ABS IgG, and Mastablot TP IgG) were included in the analysis to rule out uncertainties of TP-PA test performance in baboon sera. Mastafluor FTA-ABS IgG and Mastablot TP IgG were specifically added to cover different *T. pallidum* antigens than the rapid TTs Espline TP and Syphilitop Optima (TmpA; see Material and Methods). Interestingly, sensitivity, specificity, and the corresponding positive

and negative predictive values were lower in Serodia TP-PA when compared to all other TTs, excluding the Syphilitop Optima, which had a higher sensitivity, but weaker specificity (Table 4). This was in contrary to the TP-PA manufacturer's information about performance characteristics in human test sera (Table 1) and also when compared to FTA-ABS and immunoblot IgG results of human samples [21, 37]. In our experience the interpretation of the gelatin TP-PA requires a certain level of training and serum pre-absorption in baboon samples. This was achieved by incubating the test serum with non-sensitized particles so that unspecific binding factors were pre-absorbed. Another difficulty of the test was related to the endpoints, which can differ over time. Despite those difficulties, Serodia TP-PA has an advantage in that the readability was made by the naked eye and may be operated in resource poor laboratory settings. Also, it was the only TT that can be used for semi quantitative titer quantification. Validation of dried blood spots with a Serodia TP-PA assay for external quality assurance of *T. pallidum* serology as published elsewhere [38], provides an interesting outlook for the confirmation of screening test results, e.g., from the use of Espline TP in remote areas at the wildlife-human interface.

Both rapid TTs that could be used for screening, the Espline TP and Syphilitop Optima, were easy to perform. Results are reported within 15 min and especially the Espline TP comes in a handy cassette format. Both tests require only 25 μl of sample material. According to the manufacture's description serum and plasma samples can be used for Espline TP, while Syphilitop Optima must not use plasma samples. Comparing Espline TP to the Serodia TP-PA in serum samples of baboons, Espline TP had higher sensitivity (97.7 vs. 91.3%) and nearly the same specificity (96.0 vs 95.5%; Table 3). When the test performances are compared to the consensus of infection status (Table 2), sensitivity is 100% in both screening tests, but Syphilitop Optima achieved only 88.0% specificity compared to the Espline TP with 100% (Table 4). The reason for these differences remains uncertain but might be promoted by protein variations of the coated filter membrane. Espline TP uses at least one additional antigen (Tp 15) that is not included into Syphilitop Optima. Also, proteins (Tp 47 and Tp15-17) are used to coat two different areas of the membrane in the Espline TP, but they are combined in one field on the reaction membrane of the Syphilitop Optima. Unfortunately, there was no information available about the quantity of protein coated to the membrane in Syphilitop Optima. The Espline TP uses a combination of alkaline and non-phosphatase labeled TP recombinant antigens. Due to the lack of information, it was not possible to compare the tests in that aspect, but differences may have an influence on the binding affinity of simian *T. pallidum* antibodies.

When compared to the consensus of infection status (Table 4) both Mastafluor FTA-ABS IgG and Mastablot TP IgG have higher sensitivity and specificity and thus can be recommended as a confirmatory test in baboons. However, it may be noted, that in human infection FTA tests are no longer recommended for the diagnosis of syphilis [22,39], which should make Mastablot TP IgG the preferred option.

In the context of the performance characteristics that are reported by the manufacturers (Table 1), Serodia TP-PA had a slightly weaker sensitivity (97.7 vs. 100%) and reduced specificity (92.0 vs. 100%, Table 4). The Espline TP rapid test had nearly identical sensitivity and specificity as indicated by the manufacturer, whereas Syphilitop Optima had an equal sensitivity (100%) with a reduced specificity value, 88% vs. 95% as communicated by the manufacturer. Future studies may benefit from heat pre-treatment of serum samples to reduce interference caused by complement proteins and unspecific binding of antibodies. Heat pre-treatment was not part of any manufacturer's protocol of the tests that were used in this study.

Although the quantification of antibodies may not be of interest for disease prevalence studies in wild baboons, it may be an important tool for characterizing simian infection. The finding that severe genital-ulcerated baboons had significant higher anti-*T. pallidum* antibody

titers than clinically non-affected animals (CNA vs. SEV, $p < 0.0001$; Fig. 1) or those with less severe genital ulceration (CNA vs. MOD and INI vs. SEV, both $p \leq 0.05$; Fig. 1) is consistent with what can be expected from the course of infection. The rating of chronicity of infection in baboons at Lake Manyara National Park was based on gross-pathology and histological examination of skin tissue samples [18].

Treponemal tests as a screening tool for non-human primate infection

The use of a NTT for the initial screening in the traditional algorithm in human infection is to avoid the detection of previously treated and non-active cases [40]. NTTs are known to produce a higher percentage of false positives [41] and test performance data of the NTTs as they are reported in Table 4 need to be interpreted with caution since it is neither known when an individual was infected nor how long anti-cardiolipid antibodies can be found in the due course of infection in wild baboons.

The decision to use and recommend a TT as a screening test for *T. pallidum* infection in NHPs was based on the following three reasons and is in accordance with the current European Guidelines on the Management of Syphilis [23]. First, wild baboons are rarely treated and once infected, treponemal clearance may be an exception rather than the norm. Second, there is a paucity of data on cross-reactivity of proteins derived from human *T. pallidum* strains with antibodies against the simian strain in baboons. Lastly since the majority of baboons were chronically infected, we had reason to belief that a number of these chronically infected baboons were non-reactive in NTTs, as it was described in untreated human syphilis infection [25–27]. However, while a lifelong anti-*T. pallidum* antibody titer in baboons provides a most useful readout for the identification of a disease hot spot that offers the possibility for simian-human infection, therapeutic interventions in wild NHPs, as it is already conducted in baboons at Gombe Stream National Park in Tanzania (Collins et al. pers. communication) may benefit from the use of the traditional algorithm since NTTs may allow the differentiation of active and inactive infection.

It is generally believed that yaws has no animal reservoir. Until identical *T. pallidum* strains are found circulating in nonhuman primates and humans in their natural environment this understanding cannot change. Yet, to this end, more research is needed before nonhuman primates can definitely be ruled out to serve as a natural reservoir for human infection. We have only recently begun to explore the range of nonhuman primate infection in Africa. Because the human-livestock-wildlife interface is constantly growing, the potential for inter-species transmission increase significantly. It is also possible that simian strains do naturally infect humans but do not cause clinical manifestations, as it is the case in Guinea baboons (*Papio papio*) in Senegal; or it may be that at least the East African simian strains cause genital ulceration in humans and may therefore not be diagnosed as yaws based on their genetics. Clearly, further research is needed before any answers can be given and serological surveys are an important tool to support these investigations and to complete our picture of *T. pallidum* infection in humans and nonhuman primates.

Based on the outcome of this study we propose an algorithm for the screening of wild **non-treated** NHP populations (Fig. 2). The algorithm aims to identify *T. pallidum* infection in wild baboons and other NHPs and may complement the current yaws eradication campaign [42].

Conclusion

All tests used in this study provided reliable results to detect anti-*T. pallidum* antibodies in serum of baboons. We therefore favor hypothesis A, which suggests that commercially available serological tests are able to detect simian anti-*T. pallidum* IgM and IgG in serum samples of baboons, with the exception of IgM class anti-*T. pallidum* antibodies. It would be necessary

to examine more animals in the initial stage of infection in order to test this part of the hypothesis, something that is difficult to achieve since the time of infection in wild baboons in general is not known. While NTTs may help to plan treatment and control of infections in baboons, TTs are most useful to screening non-treated baboon population for the presence of *T. pallidum*. Hypothesis B was partly rejected because some serological tests were not equally reliable in their sensitivity and specificity in baboon samples compared to human serum samples.

For screening purposes, the immunochromatography based Espline TP test provided the highest sensitivity and specificity values and in addition had the most suitable format for use in the field. For confirmation, the treponemal test Mastablot TP IgG had the best performance characteristics and is therefore recommended as a gold standard. Serodia TP-PA was able to quantify antibodies against *T. pallidum* in baboons and results were consistent with the chronicity of infection. Based on this study a testing algorithm for the screening of NHP populations for *T. pallidum* infection is proposed, which may help future yaws eradication campaigns or wildlife management to identify baboons as a potential reservoir for human yaws infection.

Supporting Information

S1 Table. Individual-based dataset on clinical manifestations, molecular and serological test results.
(XLSX)

S2 Table. Comparison of NTT results from serum and plasma samples of baboons.
(XLSX)

S1 Checklist. STARD checklist for reporting of studies of diagnostic accuracy *(Version January 2003).*
(DOC)

Acknowledgments

We thank Tanzania Wildlife Research Institute, especially J. Keyyu; Tanzania National Parks, especially I. A. V. Lejora; Lake Manyara National Park (LMNP) headquarter staff as well as the Tanzania Commission for Science and Technology for their support to sample baboons at LMNP. For assistance, we thank E. Kiwele, K. Mollel, D. M. Kambarage, R. A. Muhala, and P. Wambura. A. Wehrend supervised the PhD study of SK from which sample material originates. S. Wienstroth is thanked for technical support in the laboratory. K. Mätz-Rensing provided negative control samples from the German Primate Center. ALL. DIAG S.A.S., T. Lamy is thanked to support the study with one free kit of Syphilitop Optima tests.

The reviewers are thanked for their comments and suggestions, which improved the manuscript.

Author Contributions

Conceived and designed the experiments: SK HL. Performed the experiments: SK FD SF. Analyzed the data: SK SF HL. Contributed reagents/materials/analysis tools: SK SF. Wrote the paper: SK FD EKB SF HL. Field immobilization of baboons: SK EKB.

References

1. Kapembwa M. Endemic treponematoses. In: Cook GC, Zumla AI, editors. Manson's Tropical Diseases. Philadelphia: Saunders-Elsevier; 2009. pp. 1139–1148.

2. Ho EL, Lukehart SA. Syphilis: using modern approaches to understand an old disease. J Clin Invest. 2011; 121: 4584–4592. doi: 10.1172/JCI57173 PMID: 22133883
3. Brinkman MB, McGill MA, Pettersson J, Rogers A, Matejkova P, Smajs D, et al. A novel *Treponema pallidum* antigen, TP0136, is an outer membrane protein that binds human fibronectin. Infect Immun. 2008; 76: 1848–1857. doi: 10.1128/IAI.01424-07 PMID: 18332212
4. McGill MA, Edmondson DG, Carroll JA, Cook RG, Orkiszewski RS, Norris SJ. Characterization and serologic analysis of the Treponema pallidum proteome. Infect Immun. 2010; 78: 2631–2643. doi: 10.1128/IAI.00173-10 PMID: 20385758
5. Carlson JA, Dabiri G, Cribier B, Sell S. The immunopathobiology of syphilis: the manifestations and course of syphilis are determined by the level of delayed-type hypersensitivity. Am J Dermatopathol. 2011; 33: 433–460. doi: 10.1097/DAD.0b013e3181e8b587 PMID: 21694502
6. Lafond R, Lukehart S. Biological Basis for Syphilis. Clin Microbiol Rev. 2006; 19: 29–49. PMID: 16418521
7. Sun R, Lai DH, Ren RX, Lian S, Zhang HP. *Treponema pallidum*-specific antibody expression for the diagnosis of different stages of syphilis. Chin Med J. 2013; 126: 206–210. PMID: 23324264
8. Harris A, Rosenberg AA, Riedel LM. A microflocculation test for syphilis using cardiolipin antigen. J Vener Dis Inf. 1946; 27: 169–174. PMID: 21003298
9. Portnoy J, Brewer JH, Harris A. Rapid plasma reagin card test for syphilis and other treponematoses. Public Health Rep. 1962; 77: 645–652. PMID: 14488129
10. Harper K, Knauf S.*Treponema pallidum* infection in primates: clinical manifestations, epidemiology, and evolution of a stealthy pathogen. In: Brinkworth JF, Pechenkina K, editors. Primates, pathogens, and evolution. New York: Springer; 2013. pp. 189–219.
11. Harper KN, Fyumagwa RD, Hoare R, Wambura PN, Coppenhaver DH, Sapolsky RM, et al. *Treponema pallidum* Infection in the wild baboons of East Africa: distribution and genetic characterization of the strains responsible. PLoS ONE. 2012; 7: e50882. doi: 10.1371/journal.pone.0050882 PMID: 23284649
12. Zobaníková M, Strouhal M, Mikalova L, Cejkova D, Ambrozova L, Pospisilova P, et al. Whole genome sequence of the *Treponema* Fribourg-Blanc: unspecified simian isolate is highly similar to the yaws subspecies. PLoS Negl Trop Dis. 2013; 7: e2172. doi: 10.1371/journal.pntd.0002172 PMID: 23638193
13. Fribourg-Blanc A, Mollaret HH, Niel G. Serologic and microscopic confirmation of treponemosis in Guinea baboons. Bull Soc Pathol Exot Filiales. 1966; 59: 54–59. PMID: 5333741
14. Smith JL, David NJ, Indgin S, Israel CW, Levine BM, Justice J, et al. Neuro-ophthalmological study of late yaws and pinta. II. The Caracas project. Br J Vener Dis. 1971; 47: 226–251. PMID: 4936861
15. Knauf S, Liu H, Harper KN. *Treponemal* infection in nonhuman primates may serve as a potential reservoir for human yaws. Emerg Infect Dis. 2013; 19: 2058–2060. doi: 10.3201/eid1912.130863 PMID: 24274094
16. Fribourg-Blanc A, Mollaret HH. Natural treponematosis of the African primate. Primates Med. 1969; 3: 113–121. PMID: 5006024
17. Levrero F, Gatti S, Gautier-Hion A, Menard N. Yaws disease in a wild gorilla population and its impact on the reproductive status of males. Am J Phys Anthropol. 2007; 132: 568–575. PMID: 17274014
18. Knauf S, Batamuzi EK, Mlengeya T, Kilewo M, Lejora IA, Nordhoff M, et al. *Treponema* infection associated with genital ulceration in wild baboons. Vet Pathol. 2012; 49: 292–303. doi: 10.1177/0300985811402839 PMID: 21411621
19. Leslie DE, Azzato F, Karapanagiotidis T, Leydon J, Fyfe J. Development of a real-time PCR assay to detect *Treponema pallidum* in clinical specimens and assessment of the assay's performance by comparison with serological testing. J Clin Microbiol. 2007; 45: 93–96. PMID: 17065262
20. Liu H, Rodes B, Chen CY, Steiner B. New tests for syphilis: rational design of a PCR method for detection of *Treponema pallidum* in clinical specimens using unique regions of the DNA polymerase I gene. J Clin Microbiol. 2001; 39: 1941–1946. PMID: 11326018
21. Binnicker MJ, Jespersen DJ, Rollins LO. *Treponema*-specific tests for serodiagnosis of syphilis: comparative evaluation of seven assays. J Clin Microbiol. 2011; 49: 1313–1317. doi: 10.1128/JCM.02555-10 PMID: 21346050
22. French P, Gomberg M, Janier M, Schmidt B, van Voorst Vader P, Young H. IUSTI: 2008 European guidelines on the management of syphilis. Int J STD AIDS. 2009; 20: 300–309. doi: 10.1258/ijsa.2008.008510 PMID: 19386965
23. International Union Against Sexually Transmitted Infections (IUSTI). European Guidelines on the Management of Syphilis. IUSTI. 2014; Available: http://www.iusti.org/

24. Sidana R, Mangala HC, Murugesh SB, Ravindra K. Prozone phenomenon in secondary syphilis. Indian J Sex Transm Dis. 2011; 32: 47–49. doi: 10.4103/0253-7184.81256 PMID: 21799578
25. Harner RE, Smith JL, Israel CW. The FTA-ABS test in late syphilis. A serological study in 1,985 cases. JAMA. 1968; 203: 545–548. PMID: 4865707
26. Hart G. Syphilis tests in diagnostic and therapeutic decision making. Ann Intern Med. 1986; 104: 368–376. PMID: 3511822
27. Reeves RR. Unreliability of syphilis screening tests. OTO-HNS. 2000; 122: 156.
28. Peeling RW, Ye H. Diagnostic tools for preventing and managing maternal and congenital syphilis: an overview. Bull World Health Organ. 2004; 82: 439–446. PMID: 15356937
29. Bosshard PP. Usefulness of IgM-specific enzyme immunoassays for serodiagnosis of syphilis: comparative evaluation of three different assays. J Infect. 2013; 67: 35–42. doi: 10.1016/j.jinf.2013.03.011 PMID: 23542782
30. Salazar JC, Hazlett KR, Radolf JD. The immune response to infection with *Treponema pallidum*, the stealth pathogen. Microbes Infect. 2002; 4: 1133–1140. PMID: 12361913
31. Teunis PF, van Eijkeren JC, Ang CW, van Duynhoven YT, Simonsen JB, et al. Biomarker dynamics: estimating infection rates from serological data. Stat Med. 2012; 31: 2240–2248. doi: 10.1002/sim.5322 PMID: 22419564
32. Hofmann H. Lyme borreliosis—Problems of serological diagnosis. Infection. 1996; 24: 470–472. PMID: 9007597
33. O'Neill P, Nicol CS. IgM class antitreponemal antibody in treated and untreated syphilis. Br J Vener Dis. 1972; 48: 460–463. PMID: 4568465
34. Egglestone SI, Turner AJ. Serological diagnosis of syphilis. PHLS Syphilis Serology Working Group. Commun Dis Public Health. 2000; 3: 158–162. PMID: 11014025
35. Young H. Guidelines for serological testing for syphilis. Sex Transm Infect. 2000; 76: 403–405. PMID: 11141863
36. Loeffelholz MJ, Binnicker MJ. It is time to use treponema-specific antibody screening tests for diagnosis of syphilis. J Clin Microbiol. 2012; 50: 2–6. doi: 10.1128/JCM.06347-11 PMID: 22090405
37. Sena AC, White BL, Sparling PF. Novel *Treponema pallidum* serologic tests: a paradigm shift in syphilis screening for the 21st century. Clin Infect Dis. 2010; 51: 700–708. doi: 10.1086/655832 PMID: 20687840
38. Smit PW, van der Vlis T, Mabey D, Changalucha J, Mngara J, Clark BD, et al. The development and validation of dried blood spots for external quality assurance of syphilis serology. BMC Infect Dis. 2013; 13: 102. doi: 10.1186/1471-2334-13-102 PMID: 23442198
39. Workowski KA, Berman S. Sexually transmitted diseases treatment guidelines. MMWR. 2010; 59: 1–110.
40. Pastuszczak M, Wojas-Pelc A. Current standards for diagnosis and treatment of syphilis: selection of some practical issues, based on the European (IUSTI) and U.S. (CDC) guidelines. Postepy Dermatol Alergol. 2013; 30: 203–210. doi: 10.5114/pdia.2013.37029 PMID: 24278076
41. Nandwani R, Evans DT. Are you sure it's syphilis? A review of false positive serology. Int J STD AIDS. 1995; 6: 241–248. PMID: 7548285
42. Maurice J. WHO plans new yaws eradication campaign. Lancet. 2012; 379: 1377–1378. PMID: 22509526
